# Supplementary material for: Perspectives and experiences with sleep and recovery among women receiving buprenorphine for opioid use disorder
Source: PLOS Ment Health. 2025 Apr 8;2(4):e0000250. doi: 10.1371/journal.pmen.0000250 (PMC12393156; doi:10.1371/journal.pmen.0000250)
Supplement: S1 Text — (DOCX) [file pmen.0000250.s001.docx]

**S1 Interview Guide**

**SECTION A: Intersection of sleep with OUD treatment and recovery**

**Theme 1: Beliefs and attitudes toward sleep while in OUD Treatment and recovery**

1. How do you define ‘healthy sleep’?
2. How important is sleep to you?
3. What happens if you don’t get enough sleep?
   1. PROBES: impair ability to think, react, cope with daily events
4. How does your sleep impact how your buprenorphine works for you?
5. How else does your sleep impact your treatment for opioid use disorder?
6. What role does sleep play in your recovery?

**Theme 2: Multi-level barriers and facilitators to healthy sleep**

1. What gets in the way of you getting a good night’s sleep?
   1. PROBES: daily life (e.g., work strain, mental health, nightmares, physical health conditions, energy drinks), family and relationships (e.g., caregiving, social support, household duties, others’ beliefs about sleep, stress, partner), community (e.g., neighborhood noise, perceived safety, violence)
2. What helps you get a good night’s sleep?
   1. PROBES: daily life (e.g., work strain, mental health, nightmares, physical health conditions, energy drinks), family and relationships (e.g., caregiving, social support, household duties, others’ beliefs about sleep, stress, partner), community (e.g., neighborhood noise, perceived safety, violence)
3. (First, ask if they have been pregnant or postpartum before) How has pregnancy or being postpartum (the months to a year after pregnancy) impacted your sleep?
4. How has your sleep changed, if at all, since you started treatment for opioid use disorder?
5. How has your sleep changed, if at all, over the course of your recovery?

**Theme 3: Medications and sleep**

1. What thoughts do you have about the role buprenorphine has played in your sleep health?
2. What medications, if any, have you ever tried in the past to help you with sleep?
3. How did these medications, if any, help your sleep?
   1. PROBES: What liked, did not like about these medications (e.g., side effects)?
4. Would you be interested in taking a medication to help with your sleep prescribed by your opioid use disorder treatment provider? Why or why not?
5. If you were to take a medication for sleep, how would you know if it’s working for you?
6. What would a medication for sleep have to do or not do, for you to like it and continue taking it?

**SECTION B: Patient insight into success and acceptability of interventions targeting sleep health**

**Theme 4: Preferences for sleep interventions**

1. What, if any, strategies on how to get better sleep have you heard?
2. Where have you gotten this information?
   1. PROBES: medical provider, social media, friends, family, magazines, TV
3. Have any of these strategies worked for you? Why or why not?

*This is a great list of strategies, here are some additional strategies that have been suggested for good sleep.*

[RA provides handout of CBT-I highlights: sleep habits, stimulus control, sleep hygiene, cognitive restructuring]

1. If you were to try some of these strategies, which do you think would be the hardest for you to do? Why?
2. In the past, have you ever worked with anyone, like a therapist, on strategies like these?
   1. If YES: What did you like or not like about it?
   2. If NO: If you have you ever wanted to work with someone on your sleep, what got in the way of you doing so?
3. In the past, have you ever worked with a therapist in a virtual or telehealth format?
   1. If YES: What did you like or not like about it?
   2. If NO: Have you ever met with another type of healthcare professional in a virtual or telehealth format? What did you like or not like about it?
4. If you were to work on these sleep strategies, would you prefer to do it in person, virtually by video chat, or virtually by phone? Why?
5. What barriers might you face with in-person sessions? What are the benefits?
6. What barriers might you face with virtual sessions (video and/or phone)? What are the benefits?

*There are sleep health programs that can be completed with a therapist virtually which can be tailored to how you sleep as recorded in your daily sleep diary.*

1. How much interest would you have in doing a sleep program like that? On a scale of 1 to 10, with 1 being not interested at all and 10 being extremely interested? Why?
2. If you were to engage in a sleep health program like this, about how many sessions would you want to engage in? And how long each session?
   1. PROBES: What is the minimum number of sessions you think would be helpful to you? What is the maximum number of sessions you would participate in? What is the minimum and maximum amount of time you would want to spend in the sessions?
3. Do you think a sleep program like this would work for you? Why or why not?
4. What would worry you or get in the way of you being able to do a sleep program like that?
   1. PROBES: not able to keep daily sleep diary, video/phone not working, wifi, distrust of therapy, distrust of technology
5. In your opinion, what would make a sleep program like that successful to you?
   1. PROBES: improved sleep health, improved other health outcome
6. In your opinion, what would make a sleep program like that be something you would recommend to others in opioid use disorder treatment?
