# Supplementary material for: Perspectives and experiences with sleep and recovery among women receiving buprenorphine for opioid use disorder
Source: PLOS Ment Health. 2025 Apr 8;2(4):e0000250. doi: 10.1371/journal.pmen.0000250 (PMC12393156; doi:10.1371/journal.pmen.0000250)
Supplement: Attachment — Submitted filename: PLOS Mental Health Revise and Resubmit.docx [file pmen.0000250.s002.docx]

Perspectives and experiences with sleep and recovery among women receiving buprenorphine for opioid use disorder

Reviewer Response Document

To Editorial Staff of PLOS Mental Health,

We would like to thank the reviewers and editor of the journal for their consideration of our manuscript. Further, we deeply appreciate the opportunity to make revisions and resubmit this work. We believe that the revisions as outlined in this response document and in the updated manuscript improve the rigor and relevance of our work.

We have aimed to be as responsive as possible to feedback that emerged during the peer review process (see below), but if any further information is needed, please do not hesitate to contact us.

Sincerely,

Michelle Eglovitch

Anna Beth Parlier-Ahmad

Alison J. Patev

Brenna Cook

Chengxian Shi

Stephanie Violante

Joseph M. Dzierzewski

Morgan H. James, PhD

Caitlin E. Martin, MD, MPH

**Comments to the Author**

**Reviewer #1:**

This manuscript describes a crucial research topic: insomnia in drug recovery. Indeed, once this topic has been reviewed, it will provide information needed by the academic literature on considering perspectives of users of drugs since they are the one experience it and felt the need to recover. they are expert of their problems and may provide professionals other ways of helping them to recover. Focusing on the methodology where the authors say that the design is mixed, but it is noticeable that it is more qualitative. I encourage them to develop the quantitative aspect as well so that it fulfills the elements of a mixed approach.

- *We appreciate this comment from the reviewer. The following sentences have been added on page 11 to give context to the quantitative analysis: For example, as sleep health behaviors naturally arose from the qualitative data, we chose to analyze quantitative data around such behaviors as collected in the survey battery. Descriptive statistics and univariate analyses were produced for demographic, psychosocial, clinical, and these self-reported sleep health behavior variables using SPSS version 28.*

**Reviewer #2:**

This is a valuable research study that contributes to the advancement of sleep medicine knowledge. However, to further enhance its impact, consider addressing the following points:

Methodology:

Scale Validation: Please clarify whether the Scale for Sleep Behaviors was validated for the specific population and context of this study.

- *We appreciate this request for clarification from the reviewer. The scale used to measure sleep behaviors was not a validated instrument. We developed the questions using the clinical expertise of the study team, as we intended to explore the prevalence of such behaviors rooted in clinical experience. This context has been added on page 8: The team intended to explore the prevalence of such behaviors as rooted in the clinical experiences; as such, these items were created by the research team, who have clinical and research expertise in the intersection between sex and gender, sleep, and OUD.*

Interview Guide: Providing the interview guide would enhance transparency and reproducibility.

- *We agree that providing the interview guide would be helpful. This has been added as supplementary material.*

Interview Methods: While Zoom interviews were mentioned, please specify if other modes (in-person or phone calls) were used. If so, please detail the recording and transcription processes for these different methods.

- *We recognize the need for clarity on interview methods. The following sentence has been added on page 10: For in-person and phone interviews (n=3), an encrypted audio recording phone application was used, and the research assistant transcribed the interview.*

Data Analysis: Please provide a more detailed explanation of the statistical analyses performed on the quantitative data.

- *The following context has been added on page 11: Descriptive statistics and univariate analyses were produced for demographic, psychosocial, clinical, and these self-reported sleep health behavior variables using SPSS version 28 (IBM SPSS Statistics for Mac, 2023).*

Data Sharing: While the transcripts were anonymized, sharing the coded transcripts (without personal identifiers) could further contribute to the transparency and replicability of the study.

- *Due to the sensitive nature of the transcripts covering topics of illicit substance use, as well as potential identifiers within the content of the transcripts themselves, we have decided not to share coded transcripts. However, the list of themes and subthemes provided in Table 3 (page 17), which include all utilized codes, can assist in contributing to study replicability.*

By addressing these points, you can strengthen the rigor and impact of your research.

**Reviewer #3:**

Thank you for the opportunity to review this manuscript. The topic is both exciting and unique, addressing an understudied area of sleep health in women undergoing OUD treatment with MOUD. The manuscript effectively combines both quantitative and qualitative methodologies, providing a rich and multifaceted understanding of how sleep impacts recovery outcomes. However, there is much room for improvement in the manuscript. Please check my specific and overall comments.

Abstract: The abstract provides a good foundation but would benefit from a few enhancements to strengthen its clarity and impact. I suggest incorporating more specific findings in the results section. Additionally, clarifying the effects of OUD medication on sleep quality, particularly any challenges or benefits reported by participants, would enrich the interpretation. Also, a policy-level recommendation would offer a meaningful contribution to policy discussions.

- *We appreciate the reviewers request for policy recommendations. Given our teams expertise in research and clinical work and the formative nature of this study, an expert-level policy recommendation is outside the scope of our paper. However, policymakers can review use our research and clinical recommendations, such as the consideration of upstream factors in the development, study, and evaluation of sleep interventions as outlined on page 21, to inform policies.*
- *In addition, more results have been added to the abstract on page 2.*

Introduction
Lines 97–99: Although comorbid psychological conditions are highlighted as factors in dropout rates, further clarification on the prevalence and specific impact of sleep disorders as comorbidities in previous research would strengthen the connection to this study's focus.

- *The following sentence and citations have been added on page 3: Sleep issues are highly prevalent among people receiving MOUD; one systematic review found prevalence rates of sleep disorders in this population ranged from 19-41%, with one recent study finding clinically significant insomnia symptoms in up to 60% of the sample (7,8).*

Lines 101–110: The link between poor sleep and substance use recurrence could be expanded to include more recent evidence or direct statistics on how sleep issues impact relapse, specifically within the OUD population. This would highlight the need for sleep interventions in OUD treatment.

- *To our knowledge, there are no national prevalence estimates of risk of return to use specifically among people with OUD with insomnia. However, more context and citations have been added to page 4: In addition, the consequences of poor sleep in this population are critical. For those with substance use disorders (SUDs) who are in recovery, research demonstrates that insomnia increases risk of return to use, even years into treatment (9,10).*

Lines 121–130: The justification for using a qualitative approach is sound but could be more explicit about why this approach is particularly critical for understanding patient-centered experiences in this context. Highlighting that qualitative insights can reveal treatment gaps or inform targeted sleep interventions for women would provide more substantial support.

- *We appreciate the request for more context around the importance of qualitative research. The following sentence has been added on page 5: Qualitative insights can reveal treatment gaps for this population by delving into experiences, perspectives, and motivations for engaging with the healthcare system.*

Lines 131–138: While the gap in research on sleep and OUD recovery from the patient perspective is noted, the section could more clearly state the specific need for patient-reported outcomes on sleep during OUD recovery, linking this to how it can inform therapeutic adjustments.

- *We appreciate the request for more specific linkages. The following has been added on page 5: Thus, collecting subjective experiences with sleep can be a critical first step in understanding how people on MOUD may experience sleep differently than other populations. These patient reported outcomes can help researchers and clinicians understand how sleep interventions can be developed or tailored and subsequently improve treatment in the OUD clinical setting.*

Methods
-Overall comment: The methods section is well-detailed, offering a comprehensive description of participant recruitment, data collection, and both qualitative and quantitative measures used to assess sleep, mental health, and other psychosocial factors among women in MOUD treatment. However, the study could be enriched by including statistical tests, such as a chi-squared test, to explore significant associations between clinically elevated insomnia symptoms and other demographic or psychosocial variables. This addition would provide a clearer quantitative insight into factors associated with insomnia severity

- *We appreciate this request from the reviewer. However, responses between those that did and did not have significant insomnia symptoms has already been published in another PLOS journal and is also outside of the scope of this study’s descriptive aims. The citation for the previously published research is as follows: White, A.M., Eglovitch, M., Parlier-Ahmad, A.B., Dzierzewski, J.M., James, M., Bjork, J.M., Moeller, F.G. and Martin, C.E., 2024. Insomnia symptoms and neurofunctional correlates among adults receiving buprenorphine for opioid use disorder. PloS one, 19(6), p.e0304461.*

Result
Line 277-228: There's an inconsistency in reporting the number of participants with clinically elevated insomnia symptoms. The methods section states that 54 participants reported elevated insomnia symptoms, but the results section describes only 50 participants meeting this criterion. This discrepancy suggests a possible oversight or error in participant tracking or data reporting. The researchers may have unintentionally excluded four participants or may not have updated the sample size accurately between sections. Clarifying this inconsistency would be essential to ensure the integrity and reliability of the study findings. The researchers should explain any exclusion criteria or adjustments made after the initial count to avoid confusion and provide transparency in participant selection.

- *We apologize for this oversight. In an initial draft of this paper, a different cutoff was used to identify participants with clinically significant insomnia (>=10). However, the proper cutoff is >=11; thus, the 54 was an error and the numbers have been adjusted as such.*

Line 283-284: According to the STROBE guidelines, "Sociodemographic Characteristics" should indeed refer to the full distribution of all participants, which in this study is n=78. If the researchers intend only to present characteristics of participants with clinically elevated insomnia symptoms, the table title should clarify this scope to avoid confusion. A clearer title could be: "Table 1: Sociodemographic Characteristics Among Respondents with Clinically Elevated Insomnia Symptoms." This approach maintains transparency and aligns with research reporting standards, ensuring readers understand which subset of the study population is being described.

- *We appreciate and agree with this comment from the reviewer. The title for Table 1 has been updated accordingly on page 12.*

Table 1: Also, in Table 1, there are a few discrepancies that raise questions about the data's accuracy and the methodology used. First, there is an issue with the Race category. The percentages for race among participants with clinically elevated insomnia symptoms sum to 100.9% (64.0% White, 30.0% Black, and 6.9% Other), which exceeds 100% and is not feasible, suggesting a potential calculation or rounding error. Additionally, the Employment status category also exhibits an irregularity: the number of participants classified as unemployed (50.0%) and employed (30.0%) only accounts for 80% of the total sample with elevated insomnia symptoms, leaving 20% unaccounted for, which could imply missing data or another calculation error.

- *We appreciate this reviewers attention to detail. The sample size for the Race category was correct, but there was a rounding error. 6.9% has been fixed to 6.0% in Table 1.*
- *In addition, there was no missing data for employment status, but the Disabled category was not included in the Table. We have included a row for those who indicated Disabled in the survey in Table 1.*

Line 288: "Co-morbid anxiety and depression were highly prevalent in the overall sample, with nearly half of the participants endorsing clinically significant anxiety, and almost 3/4ths of the sample endorsing clinically significant depression." Here, the authors gave a vague interpretation. The authors provide a general statement on the prevalence of anxiety and depression but do not clarify the true percentage of participants experiencing both conditions concurrently. Calculating and reporting the percentage of respondents with co-morbid anxiety and depression would offer a more precise measure of co-morbidity, allowing for clearer insight into the overlap of these symptoms within the sample.

- *We appreciate this reviewers attention to detail. We have calculated and added rates of comorbid anxiety and depression into Table 2, as well as added the following sentence on page 13: In addition, nearly half of participants in both categories endorsed co-morbid anxiety and depressive symptoms.*

Line 290-291: "The average buprenorphine dose was 24mg/day, which is within the standard range of buprenorphine dosing." The authors presented the median value in Table 2, “Buprenorphine dose (milligrams) (median [range]) 24 (4-30)”, but they interpreted it as a mean value, which is totally incorrect.

- *We appreciate this comment from the reviewer. The language has been updated on page 12: The median buprenorphine dose was 24mg/day, which is within the standard range of buprenorphine dosing.*

Discussion
-Overall comment: The discussion points out that the study is among the first to examine sleep experiences, specifically among women on buprenorphine. This could be emphasised more prominently at the start of the discussion to underscore the study’s unique contributions to both the sleep and OUD treatment fields. Framing the findings as filling a crucial gap in gender-specific research on sleep during OUD recovery would also strengthen its significance.

- *We agree that the uniqueness of this study should be emphasized. The following sentence has been reworded at the beginning of the discussion on page 19: To our knowledge, this study is among the first to explore patient-reported experiences with sleep and recovery among women receiving buprenorphine with insomnia symptoms.*

The discussion section could be significantly strengthened by incorporating more evidence to back up the study's findings, particularly regarding the implications of sleep on recovery outcomes and the specific role of MOUD in influencing sleep patterns among individuals with OUD.

- *We agree with this reviewer that the discussion could benefit from additional evidence. Additional support, including three additional citations, have been woven throughout the discussion particularly on pages 20 and 21.*

Line 96-99: The discussion mentions that sleep, anxiety, and depression are interlinked and can influence treatment outcomes in OUD. However, evidence detailing how anxiety and depression specifically mediate the impact of poor sleep on recovery would clarify this relationship. Including studies that analyse the impact of co-morbid mental health conditions on sleep quality and recovery outcomes among MO.

- *We appreciate this request for additional citations. To our knowledge, there are no studies that have looked specifically at how co-morbid diagnosed mental health issues mediate the impact between sleep quality and recovery outcomes among people on MOUD; our lab is among the first to research this critical issue. However, more evidence among SUD populations broadly has been added on page 20: For example, literature demonstrates that anxiety and sleep issues both impact the effectiveness of substance use treatment; for example, one study among treatment-seeking individuals with SUD found that mental health conditions actually mediated the relationship between severity of substance use and insomnia (40).*

Line 419-428: While the discussion suggests that sleep health is a promising target for interventions, it could be further developed by proposing specific clinical or policy recommendations. For instance, integrating sleep health assessments into routine OUD treatment or training clinicians on sleep hygiene for patients could be practical applications of this research. Expanding on the potential for multidisciplinary interventions to address sleep disturbances, particularly for women balancing caregiving and environmental stressors, would enhance the discussion’s relevance for practice.

- *We appreciate the request for more specific clinical applications. The following has been added on page 21: Strong qualitative data has the potential to directly inform sleep interventions in the clinical setting; for example, by helping inform tailored sleep assessments into regular MOUD visits, or training behavioral clinicians on sleep among women on MOUD. Further, the relationship of upstream factors to sleep in this population poses an opportunity for the development of multidisciplinary interventions integrating different clinical fields.*

Line 460-471: The manuscript would benefit from a clearer presentation of its strengths and limitations. Consider adding a dedicated bullet-point to distinguish this section.

- *We appreciate this suggestion from this reviewer. We have added a subheading for the limitations section.*

Line 473-477: The manuscript could conclude with a more definitive statement about the importance of sleep in OUD recovery, emphasising the potential benefits of addressing sleep in OUD interventions. A strong closing statement highlighting the promise of targeted, gender-responsive sleep interventions would leave a lasting impression and reinforce the study’s contribution to improving outcomes for women in MOUD.

- *We appreciate this suggestion to strengthen the conclusion. We have added the following sentence on page 23: To our knowledge, this study is among the first to evaluate sleep experiences among women on MOUD, and can help inform tailored, gender-responsive sleep interventions among this population.*
- *In addition, the following has been added on page 21: Strong qualitative data has the potential to directly inform sleep interventions in the clinical setting; for example, by helping inform tailored sleep assessments into regular MOUD visits or training behavioral clinicians on sleep among women on MOUD. Further, the relationship of upstream factors to sleep in this population poses an opportunity for the development of multidisciplinary interventions integrating different clinical fields.*

**Reviewer #4:**

It is mentioned that the study corresponds to a mixed method, it is inferred that it is sequential, but it is not explicitly stated what type of study it corresponds to.

- *The reviewer is correct, in that this study is a sequential mixed methods design as indicated on page 11: This study utilized a sequential mixed methods design, meaning that based on the qualitative findings, we selected quantitative data for analysis from our robust battery of measures.*

They mention the instruments used in the quantitative phase and state that they are valid, but do not provide specific data on their psychometric properties to justify their use in comparison with other existing instruments.

- *The GAD-7, PHQ-9, PROMIS-10, and Distress Tolerance Scale are all validated tools. The only tool not validated is the sleep health behaviors measure. We developed the questions using the clinical expertise of the study team, as we intended to explore the prevalence of such behaviors rooted in clinical experience. This context has been added on page 7.*

Some bivariate analyses can be carried out to show whether the secondary variables are related to the main one.

- *We appreciate this request from the reviewer. Bivariate analyses of the data have already been published (in another PLOS journal) and is also outside of the scope of this study’s purely descriptive aims. The citation for the previously published research is as follows: White, A.M., Eglovitch, M., Parlier-Ahmad, A.B., Dzierzewski, J.M., James, M., Bjork, J.M., Moeller, F.G. and Martin, C.E., 2024. Insomnia symptoms and neurofunctional correlates among adults receiving buprenorphine for opioid use disorder. PloS one, 19(6), p.e0304461.*

In the data analysis (interpretation) phase, there is no clear description of the data integration phase.

- *We agree that this section could benefit from some clarification. More context on data integration has been added on page 11: For example, as sleep health behaviors naturally arose from the qualitative data, we chose to analyze quantitative data around such behaviors as collected in the survey battery. Descriptive statistics and univariate analyses were produced for demographic, psychosocial, clinical, and these self-reported sleep health behavior variables using SPSS version 28 (IBM SPSS Statistics for Mac, 2023). Such statistics were generated for participants with clinically elevated insomnia symptoms (n=50) and qualitative participants (n=11). Then, qualitative data was chosen to be presented by naming subthemes and overarching themes and assigning quotes to represent the individual sub-themes.*

**Reviewer #5:**

Congratulations to authors, for this great work

Abstract
Can you add a line on when was the study done, and how you recruited the participants.

- *The following context has been added on page 2: Participants were recruited during their routine treatment visits, and enrollment occurred from February 2022 through September 2023.*

Methods

Be more specific how recruitment for semi-structured interviews was done and the settings of the interview. When and where was the interviews conducted, for how long, how many interviews was done to get the saturation point?

- *Additional context on saturation is indicated on page 6: Recruitment and conduct of interviews continued until data saturation occurred, at which point recruitment for the interview portion of the study was completed (n=11).*
- *Further, information about when and where the interviews were conducted is on page 6 (February 2022 – September 2023) and page 9 (Study staff provided participants with the option of completing interviews using Zoom, by phone, or in person in a private office space at the outpatient addiction clinic. The research coordinators (ME + SV) conducted the interviews, and at least one research assistant informally took notes during interviews to follow up for clarity or any missed questions as needed.)*
- *Context surrounding our recruitment methods is detailed on page 6: The research coordinator pre-screened potentially eligible participants by medical chart review then approached patients in the clinic upon arrival for their routine OUD visits to invite them to participate in the survey component of the study.*

Which data were obtained from the medical reports? (line 173-174)

- *We appreciate this request for clarification from this reviewer. The following context has been added on page 7: Finally, medical record data on treatment history was abstracted and entered into REDcap for all participants who consented to being part of the study.*

Are you measurement tools validated for your study population?

- *The GAD-7, PHQ-9, PROMIS-10, and Distress Tolerance Scale are all validated tools. The only tool not validated is the sleep health behaviors measure. We developed the questions using the clinical expertise of the study team, as we intended to explore the prevalence of such behaviors rooted in clinical experience. This context has been added on page 7.*

What was the objectives of your study? with respect to use of GAD-7 and PHQ-9? Why did you opt to use GAD-7 and PHQ-9 to screen for sleep pattern?

- *The objective of this study, as noted on page 5, was to explore patient experiences with sleep through the evolution of addiction into long-term recovery on MOUD. The Insomnia Severity Index (ISI) was used to screen for insomnia. The GAD-7 and PHQ-9 were used to assess anxiety and depression symptoms in this population; they were not used to assess sleep indices.*

GAD-7 and PHQ-9 screen for anxiety and depression symptoms which if I understand is not the aim of your study as per your title and introduction. Do you need to review your title and introduction to rationalize the use of these two tools?

- *The GAD-7 and PHQ-9 were measures used among a battery of measures given in this sample to describe the prevalence of other co-morbid factors that might relate to participant sleep, which is the primary objective of this study.*

Results
Your first paragraph of results, line 277-278 contradicts with Line 175-177 of the methodology part. Be specific how many were included in analysis 54 or 50?

- *We appreciate the reviewers attention to detail. The correct sample size (n=50) has been updated on page 11.*

NB: You have used a lot of tools like GAD-7 and PHQ-9 but not analyzed, what is the rationale of using a tool and not analyzed?

- *We appreciate this request for clarification from this reviewer. Univariate analyses of the GAD-7 and PHQ-9 are presented in Table 2 on page 13.*

**Reviewer #6:**

The study addresses an important and underexplored topic: the relationship between sleep issues and opioid use disorder (OUD) recovery among women. The use of a mixed-methods design is appropriate and commendable, as it captures both quantitative and qualitative data, providing a richer understanding of participants' subjective sleep experiences. The study findings have the potential to inform clinical practices aimed at supporting women with OUD by addressing sleep-related challenges.

- *We appreciate the kind comments from this reviewer.*
