# Supplementary material for: Perspectives and experiences with sleep and recovery among women receiving buprenorphine for opioid use disorder
Source: PLOS Ment Health. 2025 Apr 8;2(4):e0000250. doi: 10.1371/journal.pmen.0000250 (PMC12393156; doi:10.1371/journal.pmen.0000250)
Supplement: Attachment — Submitted filename: PLOS_Mental_Health_Revise_and_Resubmit_auresp_3.docx [file pmen.0000250.s004.docx]

Perspectives and experiences with sleep and recovery among women receiving buprenorphine for opioid use disorder

Reviewer Response Document

To Editorial Staff of PLOS Mental Health,

We would like to thank the reviewers and editor of the journal for their consideration of our manuscript. Further, we deeply appreciate the opportunity to make revisions and resubmit this work. We are grateful that the reviewers found the manuscript suitable for publication; this letter and the submitted manuscript address Reviewer 2 and 4’s minor suggestions.

Sincerely,

Michelle Eglovitch

Anna Beth Parlier-Ahmad

Alison J. Patev

Brenna Cook

Chengxian Shi

Stephanie Violante

Joseph M. Dzierzewski

Morgan H. James, PhD

Caitlin E. Martin, MD, MPH

**Reviewer #2:**

Nice revision to the previous manuscript. My suggestions are really minor.

When enrolling the participants, one of the exclusion criteria was that they were unable to complete the informed consent procedure. Does this mean these had passed the cognitive and psychiatric evaluation? If they had and eventually accepted to take part in the consent process but later declined, what was done to them afterwards.

- Nobody in the study withdrew consent; however, during the consent process participants were given explicit instructions on how they could contact the study team and officially withdraw consent at any point in the study, per an IRB approved script and consent form.

This may be minor. In the methods section line 182, you indicate 'see below'. It would be helpful if you guided the reader where to check for the said reference either specify the subheading.

- ‘See below’ has now been deleted.

Under the results section, there is a caption: 'Figure 1: Healthy Sleep Behaviors'. However there is no such a graph or any image accompanying the caption. Consider adding this.

- Per PLOS Mental Health guidelines, the image was uploaded separately as part of the submission process.

**Reviewer #4:**

The authors have generally responded satisfactorily to the suggestions. However, it is important that the methodology explicitly states how the integration of quantitative and qualitative data has been carried out.

- The following sentence has been added on page 11: *Thus, both quantitative and qualitative data are presented in the results below to provide a comprehensive picture of the study sample.*
